# Family function and adolescent altruistic behavior: the multiple mediating effects of self-affirmation and psychological resilience

**DOI:** 10.3389/fpsyg.2023.1184985

**Published:** 2023-07-20

**Authors:** Hongbo Cui, Xiaoyan Bi, Xiaowen Zhou, Weixin Zhang, Yankun Ma

**Affiliations:** ^1^School of Education, Guangzhou University, Guangzhou, China; ^2^Mental Health Education and Counseling Center, Guangzhou University, Guangzhou, China; ^3^Sun Yat-sen Memorial Secondary School, Zhongshan, China

**Keywords:** family function, altruistic behavior, self-affirmation, psychological resilience, multiple mediating effects

## Abstract

**Introduction:**

The current study aimed to explore the relationship between family function and adolescent altruistic behavior, as well as the mediating effects of self-affirmation and psychological resilience in this relationship.

**Methods:**

A survey was conducted on 972 high school students in Guangdong Province using the Family APGAR, GHQSense of Adequacy, Chinese version of Connor-Davidson Resilience Scale, and Altruistic Behavior Scale.

**Results:**

Results found that the score of psychological resilience of males was significantly higher than that of females, but the score of altruistic behavior was significantly lower than that of females. Family function had a positive predictive effect on altruistic behavior. Psychological resilience played a mediating role between family function and altruistic behavior. Self-affirmation and psychological resilience played chain mediating roles between family function and altruistic behavior.

**Discussion:**

This study indicated that family care is crucial for the development of adolescent altruistic behavior, and that it can promote the development of altruistic behavior through the enhancement of self-affirmation and psychological resilience.

## Introduction

Altruistic behavior, which refers to the behavior of individuals who voluntarily help others without expecting any rewards or benefits, is an essential aspect of social and moral development during adolescence (Batson, 2010 Kurzban et al., 2015). It reflects a positive attitude toward others, and is considered to be an important indicator of altruistic behavior and social responsibility. Increasingly, research on adolescents showed that altruistic behavior may help reduce dropouts, physical violence, bullying, and mental health problems (Zheng et al., 2021; Herne et al., 2022). Others have pointed out that altruistic behavior is a powerful predictor of children’s adjustment outcomes and academic performance (Flynn et al., 2014). Altruistic behavior also has been shown to promote self-efficacy (Patrick et al., 2018) and enhance positive self-evaluation (Fei et al., 2016). Adolescence is a critical period for individuals’ growth and development, and it is also a crucial period for shaping behavior and character traits. Thus, it is important to understand the factors that contribute to the development of adolescent altruistic behavior.

The family, as the most important primary socialization agent, plays a vital role in adolescents’ psychological and social development (Sheridan et al., 2004; Eales et al., 2021). It provides the emotional and social support that young people need to navigate through the complex developmental tasks of adolescence. Family function, which refers to the quality and effectiveness of family interaction, is a crucial factor that influences adolescent development (Miller et al., 1994; Olson and Craddock, 2019). Many researchers have sought to identify the influence of a family factor on altruistic behavior alone (Fang et al., 2004). However, the mechanisms through which family function influences adolescent altruistic behavior remain unclear. The interactive influence of family system on individual development and the complexity of its process also determines that there may be some intermediary factors between family function and altruistic behavior. Thus, the current study will explore the influence of family function on altruistic behavior of adolescents.

## Family function and altruistic behavior

According to the ecological systems theory proposed by Bronfenbrenner (1986), an individual’s development is influenced by a series of environmental systems, and the family is the most direct micro-environmental system. The development of an individual’s behavior is closely related to factors such as emotional connections and interaction patterns among family members (Ferguson et al., 2013). The family provides necessary material and emotional environments for the physical, psychological, and social development of its members, which is of great significance for their healthy growth and future development. Family function, as a comprehensive variable encompassing many family factors (Gonzalez et al., 2014), is considered to be the embodiment of emotional connections among family members, family rules, family communication, and effectiveness in dealing with external events (Olson and Craddock, 2019).

Intimacy and emotional support among family members can also promote an individual’s concern and care for others, thus fostering altruistic behavior (Mikulincer et al., 2005; Wang et al., 2020). In addition, the modeling effect of parents in the family can also influence a child’s altruistic behavior. If parents themselves are generous and caring individuals, children are more likely to learn this behavior. Conversely, in an environment where family function is poor, unhealthy interaction patterns and norms among family members, lack of emotional support and intimacy can easily lead to a lack of altruistic behavior in individuals (Fujiwara and Lee, 2012). If children do not receive attention and care in the family, and lack guidance in moral norms and values, they will have a harder time understanding and cultivating altruistic behavior (Lussier et al., 2018). Empirical research also confirmed that altruistic behavior in children is closely related to the intimacy between children and family members. For example, the study examined the relation between emotional responsiveness (using eye-tracking) and altruistic behavior (using the Dictator Game) in 4 to 5-year-old children (*N* = 96) across cultures (India and Germany), the results revealed that altruistic behavior is linked to our responsiveness to others in distress across cultures (Rajhans et al., 2016). Research has found that well-functioning family function not only has a significantly positive predictive effect on online altruistic behavior but also provides positive behavior demonstration and feedback to prevent adolescent rule-breaking and problem behavior and promote altruistic behavior (Song, 2018).

According to the Person-Context Interaction theory, the development of individual psychological behavior is influenced by both environmental and individual factors. Mensah and Kiernan (2010) believed that as an external environmental factor, family factors may affect children’s altruistic behavior through mediating processes that affect certain social cognitive abilities. While family function may affect adolescent altruistic behavior, the psychological mechanism linking these two variables remains unclear. Thus, the present study aims to elucidate the underlying mechanism of the relationship between family function and altruistic behavior.

## Self-affirmation as a mediator

Self-affirmation is a process in which individuals affirm their own value that is unrelated to the threat, in order to restore self-integrity and maintain a positive self-image when facing a threat (Steele, 1988). Self-Affirmation theory suggests that individual behavior results from the interaction between the self-system and the social system. In the self-system, the way people view themselves motivates them to behave in a certain way. Self-affirmation can enhance the clarity of an individual’s self-concept by emphasizing their values, principles, and standards, or by emphasizing their positive traits (such as kind and moral), but both can enhance an individual’s self-integrity (Crocker et al., 2008; Cohen and Sherman, 2014). Self-integrity is a sense of efficacy, which is an individual’s perception of their ability to control important outcomes. This can help enhance an individual’s sense of self-worth, thus further promoting altruistic behavior (Crocker et al., 2008). Furthermore, self-affirmation has been shown to promote greater empathy and compassion toward others, which are critical components of altruistic behavior (Exline and Zell, 2009). These theoretical models have been demonstrated in several studies. For example, researchers found that college students’ moral self-affirmation can positively predict online altruistic behavior.

This study discussion by examining the relationship between self-esteem and parental relationship in a sample of 316, the research results showed that there is a close relationship between self-esteem and parent-child relationships, and that a close parent-child relationship has a positive impact on self-esteem (Midgett et al., 2002). Negative parenting styles are negatively correlated with individuals’ self-esteem (Yang and Gu, 2011). Furthermore, research has found that higher social support is an important environmental variable that promotes individuals’ self-affirmation, enhances their confidence in overcoming difficulties, and increases their level of self-esteem (Marshall et al., 2014). In other words, individuals who have more positive evaluations of themselves and greater self-affirmation tend to have higher levels of self-esteem, to some extent predicting their level of self-esteem (Haddock and Gebauer, 2011). A family with effective functioning often provides more support and warmth among its members, suggesting that family function can promote the development of self-affirmation. Therefore, we speculate that self-affirmation may act as a mediator between family function and adolescent altruistic behavior.

## Multiple mediating effects

Psychological resilience refers to an individual’s ability to adapt and cope with stressors, adversity, and trauma (Masten, 2001). Resilience involves a complex interplay of biological, psychological, and social factors that can facilitate positive adaptation in the face of adversity (Luthar and Becker, 2010). Individuals with higher levels of resilience are better able to regulate their emotions, maintain positive relationships, and engage in adaptive coping strategies (Tugade et al., 2005). Research has found that higher levels of resilience are associated with greater altruistic behavior, including volunteering and charitable donations. A study of professional’s caregivers in Spain (Martí-Vilar et al., 2022) found that resilience is a variable prediction of altruistic behavior in health and social professionals. Resilience gets in the individual the capacity to be attentive to give answers in certain situations, being a predictor of great relevance of the altruistic behavior. The theory of Family Function suggests that the normal operation of basic functions in a family can provide physical, psychological, and economic support for family members, enhance the internal emotional connection between family members, jointly cope with stress and various stressful situations, and promote the improvement of individual psychological resilience level (Olson and Craddock, 2019). In summary, adolescents who experienced harmonious environment with a balanced family function may be more likely to develop psychological resilience, which may engage in more altruistic behavior.

On the other hand, according to the metatheory of Resilience and Resiliency (Richardson, 2002), when an individual’s inherent state of stability is disrupted by external events, they need to restructure themselves to form a new stable state. The level of the new stable state depends on the interaction between the challenges faced by the individual and various protective factors. If the influence of protective factors is greater than that of challenging factors, the individual will develop a higher level of resilience reintegration than before, leading to an enhancement of their psychological resilience. Research has found that self-affirmation helps activate individuals’ positive resources, enhance their sense of identity, enable them to view themselves and their surrounding environment from a broader perspective, maintain their overall self-integrity and positive self-image, and thus alleviate and reduce the negative impact of stress and threats to social identity (Steele, 1988; Hadden and Easterbrook, 2020). Therefore, this implies that self-affirmation may have a close relationship with the enhancement of psychological resilience. Specifically, adolescents who have a strong sense of self-worth and personal values, and who are resilient in the face of adversity, may be more likely to engage in altruistic behaviors. Currently, there is no research directly exploring the relationship between self-affirmation and psychological resilience.

## The present study

Altruism is regarded as an important quality in Chinese traditional culture and has been widely valued. Prior studies have shown that the relationship between family function and adolescent altruistic behavior is complex and influenced by psychological resilience and gender factors (Lussier et al., 2018; Cui et al., 2022). Family function may play a critical role in shaping the development of self-affirmation and psychological resilience, which in turn promote altruistic behavior. However, to the best of our knowledge, the interplay between these factors and their joint effects have not been fully explored. This study aims to fill this gap by examining the multiple mediating effects of self-affirmation and psychological resilience on the relationship between family function and adolescent altruistic behavior.

**Figure 1:**
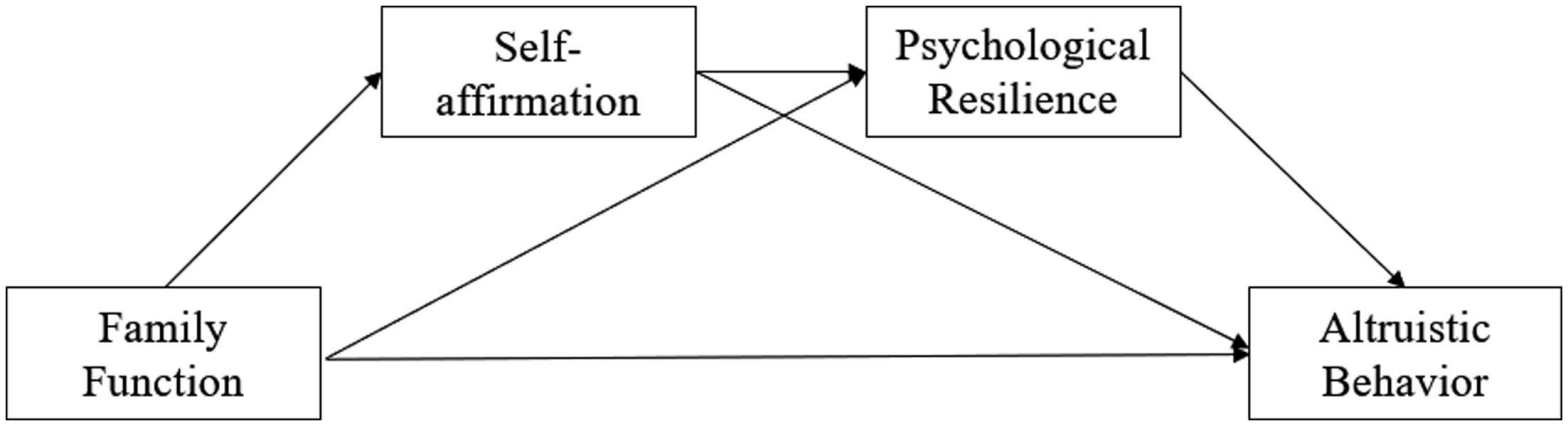
The hypothesized model.

In the present study focused on middle school students, we hypothesize that self-affirmation and psychological resilience may act as parallel mediators, with each pathway independently mediating the relationship between family function and adolescent altruistic behavior. Additionally, self-affirmation and psychological resilience may act as a chain mediator, with family function predicting self-affirmation, which in turn predicts psychological resilience, and ultimately predicts adolescent altruistic behavior. The model to be tested is presented in Figure 1.

**Figure 2:**
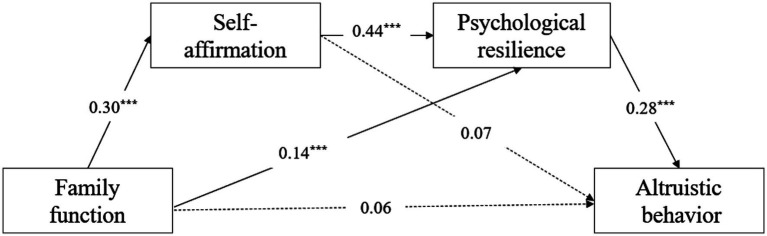
Multiple mediating effects of self-affirmation and resilience. ***p<0.001.

## Materials and methods

### Participants

This study used convenience sampling, and questionnaires of the study were gathered in March 2022 in certain normal high schools in Guangdong Province in China. After obtaining the informed consent of the school leaders and students, the researchers first explained the rules for filling in the questionnaire to the students, and then the students completed the questionnaire through mobile phones independently. A total of 1,060 questionnaires were distributed. After eliminating invalid questionnaires, 972 valid questionnaires were obtained, with an effective rate of 91.69%. The average age of the subjects is 16.74 ± 1.01 years. Among them, 400 are males (41.15%), and 572 are females (58.85%); 252 are the only children in their family (25.93%), and 720 are non-only children (74.07%); there are 338 (34.78%) students in the first year of high school and 634 (65.23%) students in the second year of high school. And 658 (67.69%) students are from urban areas, while 314 (32.30%) students are from rural areas.

## Measures

### Family APGAR

This study applied the Family APGAR which was developed by Smilkstein et al. (1982) to measure the subjects’ family function. The family APGAR consists of five items, including adaptation fitness, partnership, growth, affection, and resolve. Points of each item is assigned 0 (never), 1 (sometimes), and 2 (often). The higher total score, the better the family function. The questionnaire was modified by Lv and Gu (1995) to be suitable for Chinese. The Chinese version has been reported as a reliable tool. In this study, the Cronbach’s alpha coefficient for the scale was 0.83.

### GHQ-sense of adequacy

This study applied the General Health Questionnaire (GHQ) Sense of Adequacy which was developed by Goldberg et al. (1998) to measure the self-affirmation. The GHQ (20-item) was modified by Lihong and his coworkers (Li and Mei, 2002) for localization to be suitable for Chinese. This study selected the GHQ-Sense of Adequacy (9-item) subscale from the GHQ-20 questionnaire. The scale is scored from “Yes = 1” to “No = 0.” The higher the score, the stronger the degree of self-affirmation. The items were found to be reliable and valid in prior research (Li and Mei, 2002). The GHQ-Sense of Adequacy Cronbach’s α coefficient is 0.71 in the present study.

### Chinese version of Connor-Davidson resilience scale

This study applied the Chinese version of Connor-Davidson Resilience Scale (CD-RISC) to measure the psychological resilience. The scale initially comprised 5 factors and 25 self-reported questions (Connor and Davidson, 2003). In China, Yu et al. (2011) translated the scale into a Chinese version and instead yielded a 3-factor structure (tenacity, strength, and optimism) in a Chinese community sample. Many subsequent studies have provided evidence that the revised scale is more suitable for Chinese (Xie et al., 2016). The 25-item Likert-scale (0 = not at all, 4 = extremely true) was used to assess the resilience of the study sample with a total of 100 scores. A higher score indicates a higher level of resilience. The Chinese version of Connor-Davidson Resilience Scale Cronbach’s α coefficient is 0.93 in the present study.

### Altruistic behavior scale

The scale, prepared by Li (2008), contains five dimensions: altruistic behavior responsibility, respect and care for others, care and care for oneself, altruistic behavior performance, and self-interested behavior and concepts. There are a total of 22 items in the scale, and the Likert 7-level score from “very inconsistent” to “very consistent” is adopted. The higher the total score, the higher the altruistic level. Among them, questions 1, 2, 3, 5, 7, 8, 10, 14, and 18 are reverse scoring. The items were found to be reliable and valid in prior research (Zheng, 2013). In this study, the internal consistency of the five factors of the scale is between 0.73 and 0.81. Cronbach’s α Coefficient is 0.82.

### Data analysis and common method bias test

This study used SPSS 26.0 to perform descriptive statistics, *t*-test, and correlation analysis on the collected data, and used the PROCESS V4.0 macro program of Hayes to test and analyze mediating effects. The data collected in this study is self-reported, so the common method deviation test is required. In this study, Harman single factor test method was used for exploratory factor analysis. The results showed that 11 common factors with characteristic value greater than 1 were obtained from the factor analysis without rotation, and a total of 11 factors were greater than 1. The first factor explained 21.07% variance and less than 40% marginal value, indicating that there was no serious common method deviation in this study (Zhou and Long, 2004).

## Results

### Descriptive statistics and correlation analysis

Descriptive statistics and Pearson’s correlations for the main variables are presented in Table 1. The results show that compared with males, females have lower scores of psychological resilience (*t* = −6.26, *p* < 0.001) and higher scores of altruistic behavior (*t* = 3.21, *p* < 0.01). The results of Pearson’s correlation analysis showed that the correlation between family function, self-affirmation, psychological resilience and altruistic behavior was significant (*p* < 0.01).

**Table 1:** Descriptive statistics and correlation coefficient matrix (*N* = 972).

	Males	Females	1	2	3	4
1. Family function	5.33 ± 2.55	5.39 ± 2.69	–			
2. Self-affirmation	5.67 ± 2.18	5.76 ± 2.32	0.29***	–		
3. Psychological resilience	63.61 ± 13.78	56.12 ± 13.80	0.30***	0.54***	–	
4. Altruistic behavior	101.89 ± 8.79	103.95 ± 8.46	0.16**	0.28***	0.39***	–

**p < 0.01, ***p < 0.001.

### Regression and mediation effect analysis

According to the results of the correlation analysis in this study and the statistical preconditions of the mediation effect, further mediation effect analysis of self-affirmation and psychological resilience can be carried out (Wen and Ye, 2014). With family function as the independent variable, altruistic behavior as the dependent variable, self-affirmation and psychological resilience as the intermediary variables, the study used the bias-corrected percentile Bootstrap method in the SPSS macro program Process compiled by Hayes to analyze the mediating effect, and Model 6, which specialized in analyzing chain mediation effects, was used for testing. The Bootstrap sampling number is 5000, the confidence interval is set to 95%.

The results show that family function has a positive predictive effect on altruistic behavior (*β* = 0.20, *p* < 0.001); family function has a positive predictive effect on self-affirmation (*β* = 0.30, *p* < 0.001); family function has a positive predictive effect on psychological resilience (*β* = 0.14, *p* < 0.001); psychological resilience has a positive predictive effect on altruistic behavior (*β* = 0.28, *p* < 0.001);self-affirmation has a positive predictive effect on psychological resilience (*β* = 0.44, *p* < 0.001). Self-affirmation has no positive predictive effect on altruistic behavior (*β* = 0.07, *p* = 0.08), see Table 2.

**Table 2:** Model for regression analysis between variables (*N* = 972).

Predictor variable	Outcome variable: SA	Outcome variable: PR	Outcome variable: AB
	β (boot SE) 95%BootCI	β (Boot SE) 95%BootCI	β (Boot SE) 95%BootCI
FF	0.30 (0.03) [0.29, 0.74]	0.14 (0.09) [0.22, 0.57]	0.06 (0.11) [−0.02, 0.43]
SA		0.44 (0.10) [1.30, 1.70]	0.07 (0.15) [−0.03, 0.56]
PR			0.28 (0.05) [0.23, 0.41]
	*R*^2^ = 0.09, *F* = 26.06***	*R*^2^ = 0.31, *F* = 89.29***	*R*^2^ = 0.13, *F* = 23.01***

**p < 0.01, ***p < 0.001. FF, Family Function; SA, Self-Affirmation; PR, Psychological Resilience; AB, Altruistic Behavior.

The intermediary effect test showed that the direct effect of family function on altruistic behavior was 0.16, accounting for 39.02% of the total effect; family function affects altruistic behavior through psychological resilience, and the intermediary effect is 0.13, accounting for 31.71% of the total effect of family function on altruistic behavior; family function has an impact on altruistic behavior through the chain mediating roles of self-affirmation and psychological resilience, with an effect of 0.12, accounting for 29.27% of the total effect of family function on altruistic behavior. The mediating effect of self-affirmation between family function and altruistic behavior is not significant (The 95% BootCI is [−0.01, 0.16], including 0), see Table 3 and Figure 2.

**Table 3:** Mediation effect analysis (*N* = 972).

Model pathways	Effect size	Boot SE	95%BootCI
**Direct effect**			
FF—AB	0.16	0.11	[0.29, 0.74]
**Indirect effect**			
FF—SA—AB	0.07	0.04	[−0.01, 0.16]
FF—PR—AB	0.13	0.04	[0.06, 0.20]
FF—SA—PR—AB	0.12	0.03	[0.08, 0.17]

**p < 0.01, ***p < 0.001. FF, Family Function; SA, Self-Affirmation; PR, Psychological Resilience; AB, Altruistic Behavior.

## Discussion

Descriptive statistical results showed that boys’ scores for psychological resilience were significantly higher than those of girls. Psychological resilience is a concept influenced by gender social norms (Mackenzie et al., 2006), with deep cultural influences behind it. Generally speaking, compared to girls, parents, teachers, and society as a whole in China always hope that boys can show more independence and grow up to be a “real man” (Wang et al., 2020). This expectation of male roles enhances male independence and autonomy, both consciously and subconsciously, which leads males to always think that they should complete something independently, and their confidence in completing something also increases (Eagly and Karau, 2002; Rudman and Glick, 2021). Therefore, when facing adversity, males’ resilience often creates more coping strategies, which is part of the construction of psychological resilience. On the other hand, descriptive statistical results showed that females’ scores for altruistic behavior were significantly higher than males’. Both Eastern and Western cultures believe that females are more sensitive to the emotions of others and have stronger empathy (María et al., 2009; Wang et al., 2020). According to the Empathy-Altruism Hypothesis, this trait benefits females in discovering more needs for help and showing more altruistic behavior (Batson, 1987; Luo et al., 2013). On the other hand, Relationship Theory suggests that compared to males, females have higher expectations for developing and maintaining personal relationships with others (Susan, 1998). Females are usually more concerned with their interpersonal relationships, and are more prone to emotional distress when their social relationships are damaged. This psychological characteristic may also drive females to develop and maintain effective interpersonal relationships by showing more altruistic behavior. Under the cultural framework of China, Chinese girls are taught from an early age to be compassionate, considerate, and caring toward others; however, the requirements and subtle education for boys in Chinese culture often suggest that they should not be too concerned about details or avoid being emotional (Li et al., 2017). Therefore, Chinese girls are expected to show more altruistic behavior.

After controlling for gender and age, this study found that family function had a significant positive influence on altruistic behavior among adolescents, consistent with previous research results (Laible et al., 2004; Cui et al., 2022). In families with effective function, family members have close intimacy and higher cohesion, and parents exercise relatively less psychological and behavioral control over their children. Children in relaxed family atmospheres tend to have healthier mindsets, are better at emotional expression, and are able to perceive their own emotional changes (Gittleman et al., 1998). On the other hand, children who perceive others as benevolent and trustworthy are more likely to consider others (Mikulincer et al., 2005). Empirical research also supports this theoretical assumption: Effective family function can promote teenagers to correctly influence others’ cognition in interpersonal communication, better understand others’ emotions, and improve children’s empathy ability (Wang et al., 2020). According to the Empathy Altruism Hypothesis (Batson, 1987), when the level of opinion taking and empathy is high, adolescents are more likely to have altruistic motives and show altruistic behavior.

This study further found that family functionality affected altruistic behavior through psychological resilience. Hobfoll (2001) proposed the Conservation of Resources theory, which suggests that people actively strive to obtain, maintain, protect and cultivate valuable resources. Individuals with more resources are less susceptible to resource loss and are more likely to acquire resources. The Gain Spiral of the Conservation of Resources theory reveals that people with more resources are more likely to gain more resource benefits, and the initial resource benefits further lead to more resource benefits, thereby entering a spiral of resource gain (Hobfoll, 2001; Su, 2017). Effective family function is a valuable resource that may promote adolescents to obtain more resource gains, such as developing high levels of psychological resilience. According to the Broaden-and-Build theory of Positive Emotions (Isgett and Fredrickson, 2004), individuals with high psychological resilience can better expand their range of individual attention, cognition and action. Individuals with high levels of psychological resilience often have more positive emotions and optimistic attitudes, which further provide necessary psychological resources for adolescents to implement altruistic behavior (Tugade et al., 2005). Therefore, adolescents who grown up in a effective family atmosphere will receive higher social support, perceive harmonious and intimate family relationships, and often develop higher levels of psychological resilience, further exhibiting more altruistic behaviors. This result also coincides with the concept proposed by Social Exchange theory.

Finally, this study also found that family function could influence altruistic behavior through a chain-mediated effect of self-affirmation and psychological resilience. According to the Conservation of Resources theory and the Gain Spiral effect (Hobfoll, 2001), effective family function as a valuable resource may promote adolescents’ higher levels of self-esteem and self-worth (Yang and Gu, 2011; Marshall et al., 2014). Therefore, adolescents who grown up in a positive family function environment tend to develop higher levels of self-affirmation. The Metatheory of Resilience and Resiliency (Richardson, 2002; Feng et al., 2022) suggests that social stressors and protective factors interact to determine whether individuals can maintain internal stability. Social psychological factors are crucial in forming resilient reintegration. Based on the Motivation’s Social Cognitive theory, individuals with high self-esteem and self-worth in stressful environments tend to focus more on opportunities for improving their abilities rather than being overly concerned with setbacks, and they can maintain a positive mood, even becoming more optimistic (Dweck, 2000; Schunk and Dibenedetto, 2019). This actually implies that self-affirmation is a protective factor for psychological resilience and contributes to individuals’ resilient reintegration. Therefore, they can better perceive and understand the situation of others (Cohen and Sherman, 2014). Specifically, effective family function can facilitate the development of high levels of self-affirmation in adolescents. High levels of self-affirmation are conducive to high levels of psychological resilience, and individuals with greater psychological resilience tend to be more positive and optimistic, allowing them to accumulate more psychological resources and ultimately exhibit more altruistic behaviors. In sum, this study indicated that family care is crucial for the development of adolescent altruistic behavior, and that it can promote the development of altruistic behavior through the enhancement of self-affirmation and psychological resilience.

Firstly, parents can focus on improving family functioning by establishing a positive parent-child relationship and communication, fostering adolescents’ self-affirmation and psychological resilience, thereby promoting their altruistic behavior. Secondly, schools and educators can integrate altruistic values into educational curricula, teaching knowledge and skills related to cooperation, care, and social responsibility. Additionally, providing opportunities for students to participate in community service projects, volunteer activities, and team collaboration can cultivate their altruistic behavior. Thirdly, policymakers can develop relevant policies and initiatives to encourage altruistic behavior in the social environment. For example, implementing reward systems, scholarships, or honors to recognize and motivate adolescents’ altruistic actions. Moreover, policies can promote community engagement and the cultivation of a spirit of mutual assistance, providing more opportunities for adolescents to participate in social welfare activities. Overall, these measures and strategies, as mentioned above, can be implemented by parents, educators, and policymakers based on the research findings to promote adolescent altruistic behavior. These efforts contribute to the establishment of positive family environments, educational systems, and supportive social structures, fostering a greater number of adolescents with an altruistic spirit.

### Limitation

There are several limitations in this study. Firstly, it primarily relies on cross-sectional data, which prevents the establishment of causal relationships between variables. To gain a more comprehensive understanding, future research should consider employing longitudinal designs to explore these relationships in greater depth. Second, all variables in the study only use the subjective reporting method of individual students. More objective data need be obtained by combining multiple methods such as parents, teachers and peer evaluation to reduce the social praise effect.

## Conclusion

This study enriches our understanding of the relationships between family function and adolescent altruistic behavior. Our findings showed that the relation between family function and adolescent altruistic behavior is mediated by self-affirmation and psychological resilience. These results help us to understand that family care is crucial for the development of adolescent altruistic behavior, and that it can promote the development of altruistic behavior through the enhancement of self-affirmation and psychological resilience.

## Data availability statement

The raw data supporting the conclusions of this article will be made available by the authors, without undue reservation.

## Ethics statement

The studies involving human participants were reviewed and approved by Ethics Committee of Guangzhou University. Written informed consent to participate in this study was provided by the participants’ legal guardian/next of kin.

## Author contributions

HC conceived the study, drafted the manuscript, and took responsibility for the manuscript as a whole. YM and XB provided advice on study design and supervised the data collection. XB, WZ, and XZ participated in data collection and data analysis. All authors contributed to the article and approved the submitted version.

## Funding

This work was supported by the Guangzhou Education Scientific Planning Projects (grant number 202012558) and the Guangdong Youth Research Project (2020 WT025).

## Conflict of interest

The authors declare that the research was conducted in the absence of any commercial or financial relationships that could be construed as a potential conflict of interest.

## Publisher’s note

All claims expressed in this article are solely those of the authors and do not necessarily represent those of their affiliated organizations, or those of the publisher, the editors and the reviewers. Any product that may be evaluated in this article, or claim that may be made by its manufacturer, is not guaranteed or endorsed by the publisher.
